# Elucidating the degradation pattern of a new cold-tolerant pectate lyase used for efficient preparation of pectin oligosaccharides

**DOI:** 10.1186/s40643-021-00475-2

**Published:** 2021-12-04

**Authors:** Ling Zheng, Zilong Guo, Shengsheng Cao, Benwei Zhu

**Affiliations:** College of Food Science and Light Industry, Nanjing Tech University, Nanjing, 211816 China

**Keywords:** Pectate lyase, *Echinicola rosea*, Cold-adapted, Product analysis

## Abstract

The cold-active pectate lyases have drawn increasing attention in food and biotechnological applications due to their ability to retain high catalytic efficiency under lower temperatures, which could be helpful for energy saving, cost reduction and flavor preservation. Herein, a new cold-tolerant pectate lyase (ErPelPL1) gene from *Echinicola rosea* was cloned and heterologously expressed in *Escherichia coli*. Interestingly, ErPelPL1 retained high catalytic activity even at a low temperature (4 °C). ErPelPL1 exhibited optimal activity at 35 ℃, pH 8.0 with 1 mM of Ca^2+^. It showed high specific activity towards polygalacturonic acid (34.7 U/mg) and sodium polygalacturonate (59.3 U/mg). The combined thin-layer chromatography (TLC), fast protein liquid chromatography (FPLC) and electrospray ionization mass spectrometry (ESI-MS) results indicated that ErPelPL1 endolytically degraded pectic substances into the oligosaccharides with degrees of depolymerization (Dps) of 1–6. In conclusion, this study mainly conducted biochemical characterization and product analysis of a cold-tolerant pectate lyase. Therefore, it provides a promising enzyme candidate for food and biotechnological applications.

**Graphical Abstract:**

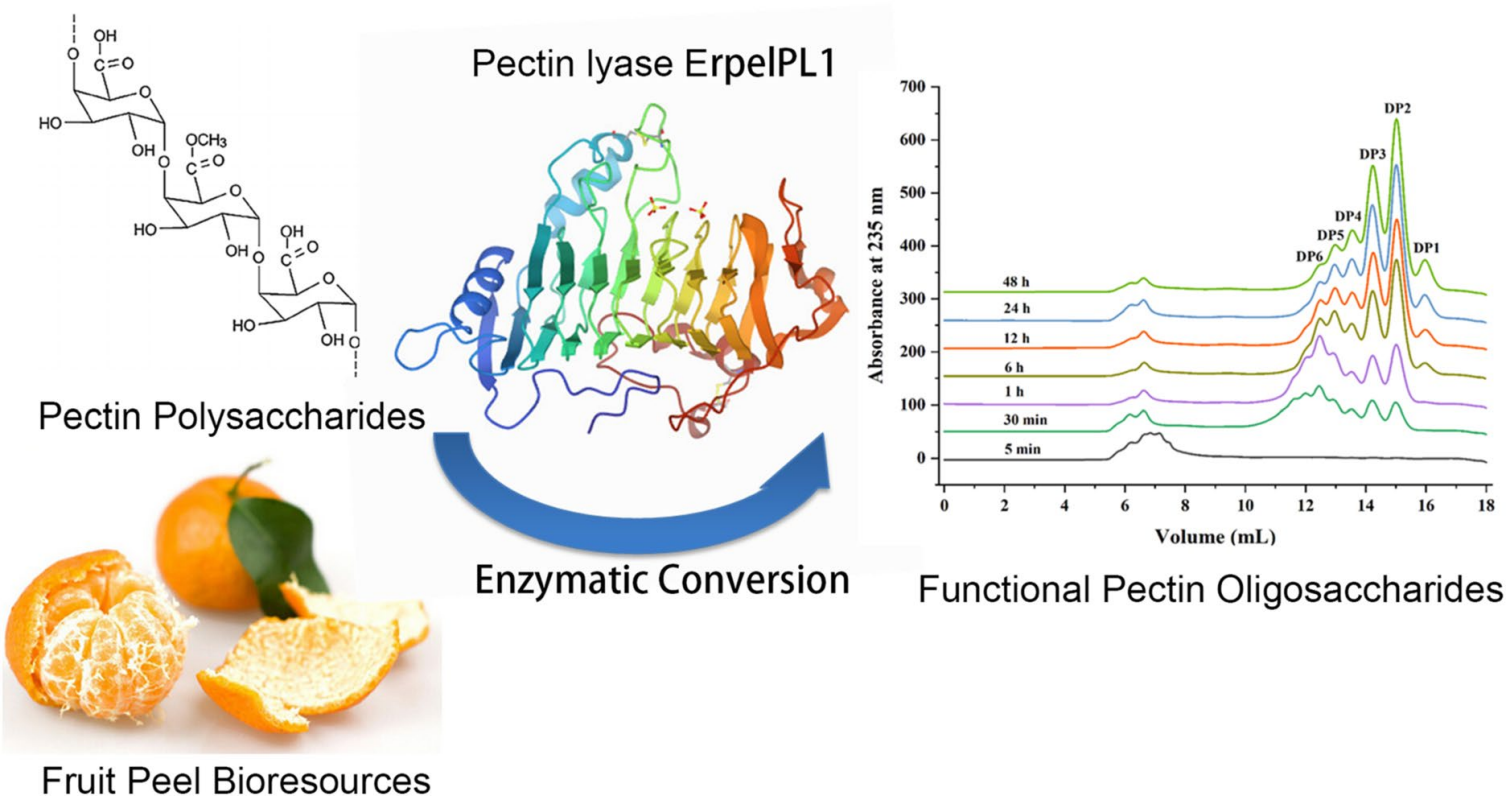

**Supplementary Information:**

The online version contains supplementary material available at 10.1186/s40643-021-00475-2.

## Introduction

Pectin, a negatively charged heteropolysaccharide, is the major component of the primary cell wall of higher plants (Kohli and Gupta 2015). It is constituted by d-galacturonic acid and other monosaccharides via α-1,4 glycosidic bonds (Zhou et al. 2017). The complex polysaccharide included a galactoglycan skeleton and some branches. The branches are composed of different monosaccharides such as rhamnose, arabinose, galactose, and xylose (Gummadi and Panda 2003; Yoder et al. 1993). To completely degrade the complex pectin, it requires the synergistic action of a group of pectinases with different substrate specificity (Bekli et al. 2019). The biological activity of pectin is often exhibited by its degradation products obtained by partial enzymatic hydrolysis (Weikert et al. 2017). Therefore, pectin oligosaccharide (POS), the degradation product of pectin, is receiving increasing attention. POS could be selectively fermented by the intestinal flora and were considered as the best choice for second-generation prebiotic factors (Olano martin et al. 2002). Numerous researches suggested that POS exhibited various physiological activities, such as prebiotic, antibacterial, anticancer and antioxidant properties. For example, Kang et al. produced the POS by irradiation from citrus and revealed it has antioxidant and cancer cell proliferation inhibition effect (Kang et al. 2006). Li et al. enzymatically degraded the orange peel into POS, and also confirmed that the POS exhibited prebiotic properties and antibacterial activity (Li et al. 2016).

In recent years, researchers have made various attempts to prepare POS, such as physical degradation, chemical hydrolysis, and enzymatic preparation. Chen et al. obtained POS by dynamic high-pressure microfluidization (Chen et al. 2013). Zhang et al. prepared POS fractions by controlled chemical degradation of citrus peel pectin (Zhang et al. 2018). Wang et al. produced POS using *Humicolainsolens* Y1-derived unusual pectate lyase (Wang et al. 2019). Compared with physical and chemical methods, enzymatic preparation had the advantages of no requirement of special equipment, good selectivity of reaction, few undesirable by-products, mild reaction conditions and high efficiency. Therefore, the enzymatic preparation of POS with good biological activity has become the focus of related field.

Approximately 10% fraction of the global enzyme market is occupied by pectinolytic enzymes, which are used in the food, paper, textile industries and biotechnology applications (Carrasco et al. 2019). These enzymes mainly include pectic hydrolases (also called polygalacturonases (PGs): endo-PG, EC 3.2.1.15, exo-PG, EC 3.2.1.67), pectin methylesterase (PME, EC 3.1.1.11), and pectinolytic lyases (Wu et al. 2020; Yadav et al. 2008; Kashyap et al. 2001; Saharan and Sharma 2019). Based on the differences of substrates and products, pectinolytic lyases were classified into pectate lyase (endo-Pel, EC 4.2.2.2 and exo-Pel, EC 4.2.2.9) and pectin lyase (PL, EC 4.2.2.10) (Liu et al. 2018). Pectate lyase (Pel) is of great significance in producing 4,5-unsaturated oligogalacturonides. The pectate lyases are specific for polygalacturonic acid (PGA) and active on low-methoxyl (LM) pectin, while pectin lyases acted on high-methoxyl (HM) pectin (Yang et al. 2020). Notably, Pel cleaved the α-1,4-glycosidic bond of the substrate molecular skeleton by β-elimination mechanism without producing highly toxic methanol (Ogawa et al. 2000; Wang et al. 2014; Jayani et al. 2005). Furthermore, cold-tolerant pectate lyase has great potential in several industrial and biotechnological applications, such as the production of bioactive material and the pretreatment of cotton fabric (Zheng et al. 2021). It can not only save energy, but also retain the labile compounds and effectively prevent the proliferation of undesired microorganisms. For example, several researchers have prepared pectin oligosaccharides, an emerging bioactive candidate, by enzymatic hydrolysis of pectin under mild conditions (Wang et al. 2019; Gómez et al. 2014). Tang et al. investigated a new cold-active and alkaline pectate lyase from Antarctic bacterium with high catalytic efficiency (Tang et al. 2019). The reduction of cloudiness and bitterness of fruit juices and grapes in the juice and wine industries should be performed at low temperatures (below 15 °C) (Carrasco et al. 2019). Therefore, psychrophilic enzymes have become a new research focus, especially in the field of industrial and biotechnological applications. Because they could not only skip heat treatment to reduce costs, but also have great benefits in maintaining the quality of products (Ramya and Pulicherla 2015). However, only three cold-active Pel-encoding genes have been reported at present, which greatly limited the application of pectate lyases (Wu et al. 2020).

Herein, a cold-tolerant pectate lyase (ErPelPL1) from *Echinicola rosea* was firstly cloned and expressed in *Escherichia coli*. The enzymatic properties and product distribution of ErPelPL1 were investigated in detail. The optimum temperature and pH for its catalytic activity were 35 °C and 8.0, respectively. The combined thin-layer chromatography (TLC), fast protein liquid chromatography (FPLC) and electrospray ionization mass spectrometry (ESI-MS) results revealed that ErPelPL1 endolytically degraded pectic substances into oligosaccharides with Dps of 1–6. This work provided new cold-tolerant pectate lyases for preparation of pectin oligosaccharides.

## Materials and methods

### Materials and strains

Polygalacturonic acid, pectin from citrus peel, and pectin from apple were purchased from Sigma-Aldrich (St. Louis, MO, USA). Sodium polygalacturonate was purchased from Shanghai Yuanye Bio-Technology Co., Ltd (Shanghai, China). D-Galacturonic acid sodium salt (purity, about 95%) was obtained from Sigma-Aldrich. All other chemicals and reagents were of analytical grade. The genome of *Echinicola rosea* JL3085^T^ harbored multi-gene polysaccharide utilization loci (PUL) systems involved in the degradation of pectin. This study used *Escherichia coli* DH5α and *E. coli* BL21 (DE3) for plasmid construction and as the hosts for gene expression, respectively.

### Sequence analysis

The theoretical molecular weight (Mw) and isoelectric point (pI) of this enzyme were calculated through the Compute *p*I/Mw tool (https://web.expasy.org/compute_pi/). Moreover, the structure domain analysis was conducted by Simple Modular Architecture Research Tool (SMART). The secondary structure analysis was performed by GOR4. The multiple sequences alignment of ErPelPL1 and other pectate lyases of polysaccharide lyase (PL) 1 family were performed by Vector-NTI (Life Technologies, Grand Island, NY). Based on the protein sequences of PL1 family, the phylogenetic tree was constructed through Molecular Evolutionary Genetics Analysis (MEGA) Program version 6.0. The homology modeling of ErPelPL1 was built by the online Protein Homology/analogY Recognition Engine V 2.0 (PHYRE) (http://www.sbg.bio.ic.ac.uk/phyre2/html/page.cgi?id=index).

### Cloning, expression, and purification

As previously reported (Zhan et al. 2020), a gene cluster for degrading pectic substances has been identified within the genome of the strain *Echinicola rosea* JL3085^T^. However, it has not been heterologously expressed and its biochemical properties have not been systematically studied. The gene of ErPelPL1 was cloned by using the primers as described in Additional file 1: Table S1. For protein expression, the gene of ErPelPL1 was ligated into pET-21a (+) expression vector and then the recombinant plasmid was transformed into *E. coli* BL21 (DE3). The recombinant strains were cultured in 100 mL Luria–Bertani (LB) broth containing 100 μL of 100 μg/mL ampicillin and agitated on a rotary shaker at 150 rpm at 37 ℃. To induce protein expression, the cells were induced by 0.2 mM isopropyl β-d-1-thiogalactopyranoside (IPTG) for 40 h (150 rpm, 18 ℃) when the optical density at 600 nm (OD_600_) was 0.4–0.6.

The cell sediments were harvested by centrifugation (3059×*g*, 4 min) and resuspended in binding buffer (10 mM Tris–HCl (pH 8.0), 300 mM NaCl, and 10 mM imidazole). The cell suspension was lysed by sonication for 30 min, and then centrifuged (10,000×*g*, 40 min) at 4 ℃ to collect the crude enzyme. Binding and elution from a high-affinity Ni-charged resin FF (Ni–NTA Sepharose) prepacked column (GeneScript, Nanjing, China) with six His-tag was performed according to the manufacturer’s instructions. The recombinant protein was eluted with 10 mM Tris–HCl (pH 8.0) containing 300 mM NaCl and 250 mM imidazole. All protein-purification steps were performed at 4 °C. According to Laemmli (1970), the eluted proteins were analyzed by sodium dodecyl sulfate polyacrylamide gel electrophoresis (SDS-PAGE), using a 12% separating gel and a 5% stacking gel. The protein concentration was determined by protein quantitative analysis kit (Beyotime Institute of Biotechnology, Nantong, China).

### Enzyme assays and enzymatic kinetics

Enzyme activity was assayed in 300 μL of reaction mixtures containing 150 μL 0.5% (w/v) substrates (pectin from apple (Pectin A), pectin from citrus peel (Pectin C), polygalacturonic acid (PGA) and polygalacturonic sodium (PG-Na)), 100 μL of 1 mM CaCl_2_, and 50 μL of purified ErPelPL1 at 35 °C for 30 min. Pectate lyase activity was determined by using the 3,5-dinitrosalicylic acid (DNS) method (Klug-Santner et al. 2006; Miller 1959). One unit of Pel activity was defined as the amount of enzyme producing 1 μmol reducing sugar per min under the above conditions (Ogawa et al. 2000). In addition, the dependence of recombinant Pels on Ca^2+^ was determined in the presence of 0–3.0 mM of CaCl_2_.

The kinetic parameters of the ErPelPL1 towards sodium polygalacturonate were evaluated by measuring the enzyme activity with substrate at different concentrations as described previously (Zhu et al. 2019). The Lineweaver–Burk plots were used to calculate the kinetic parameters *K*_*m*_ and *V*_*max*_ according to the enzyme reactions with substrate at 0.1–5 mg/mL. The ratio of *V*_*max*_ versus enzyme concentration ([E]) was used to calculate the turnover number (*k*_*cat*_) of the enzyme. All experiments were performed with three replicates.

### Biochemical characterization

The effects of temperature on the enzyme activity were determined at 25–55 ℃. To evaluate the thermostability, ErPelPL1 was incubated at 10–50 ℃ for 40 min, and the residual activities were assessed at 35 ℃ for 30 min. To investigate the optimal pH, 0.5% sodium polygalacturonate, 1 mM CaCl_2_ and the purified enzyme were incubated in different pH buffers (50 mM phosphate–citrate (pH 4.0–5.0), 50 mM NaH_2_PO_4_–Na_2_HPO_4_ (pH 6.0–8.0), 50 mM Tris–HCl (pH 7.0–9.0), and glycine–NaOH (pH 9.0–12.0)). Moreover, the pH stability was assessed by measuring residual activity after being incubated within different buffers (pH 4.0–12.0) for 24 h.

The effects of metal ions and EDTA on ErPelPL1 were performed by incubating with various metal compounds with a final concentration of 1 mM at 4 ℃ for 24 h, then determining the enzyme activity after incubation as described above. The reaction performed under standard tested conditions and the substrate blend without any metal ion was taken as the control. Furthermore, to investigate the effects of reagents, purified enzyme was incubated with 0.5% and 5% Triton X-100, urea and SDS at 4 ℃ for 24 h. Enzyme activities were expressed as percentages of that in the absence of added metal ions and reagents (100%). Chloride salts were used to study the effect of metal ions on enzyme activity. All experiments were performed with three replicates.

### Action pattern and products analysis of ErPelPL1

The reaction mixtures (1.2 mL) containing 200 μL of purified enzyme, 600 μL of sodium polygalacturonate and 400 μL 1 mM CaCl_2_ were incubated at 30 ℃ for 0–72 h. In order to elucidate the action pattern, thin-layer chromatography (TLC) was applied to analyze the degraded products of ErPelPL1 towards sodium polygalacturonate. The degradation products were separated on TLC Silica gel 60 plates (Merck-Millipore) by using *n*-butanol:formic acid:water (4:6:1). The result was visualized by heating TLC plates at 130 ℃ for 5 min after spraying with 15% (v/v) sulfuric acid in ethanol, containing 0.2% resorcinol.

In addition, the final products were also separated by fast protein liquid chromatography (FPLC). Using a Superdex Peptide 10/300GL column (GE Healthcare, USA) to separate samples and monitor at wavelength 235 nm in an ÄKTA purifier system. The column was eluted with 0.2 mol/L of NH_4_HCO_3_ at 0.5 mL/min. To further confirm the composition of the degrading products, electrospray ionization mass spectrometry (ESI-MS) was employed as follows: the supernatant (2 μL) was loop-injected to an LTQ XL linear ion trap mass spectrometer (Thermo Fisher Scientific, Waltham, MA, USA) after centrifugation. The oligosaccharides were detected in a negative-ion mode using the following settings: ion source voltage, 4.5 kV; capillary temperature, 275–300 ℃; tube lens, 250 V; sheath gas, 30 arbitrary units (AU); and scanning the mass range, 100–2000 *m/z.*

## Results and discussion

### Sequence analysis of ErPelPL1

**Fig. 1:**
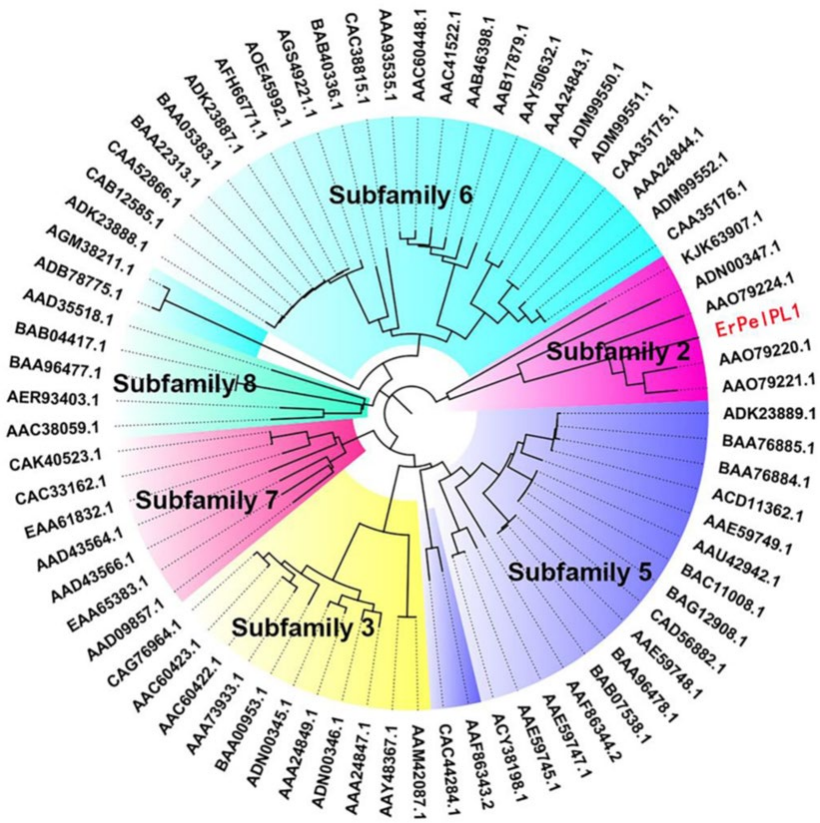
Phylogenetic analysis of ErPelPL1 with other pectate lyases of PL1 family. Different color blocks correspond to subfamilies of PL1 family, respectively

In this study, the gene of pectate lyase ErPelPL1 from *Echinicola rosea* JL3085 was firstly cloned and analyzed. ErPelPL1 included 448 amino acid residues and the theoretical molecular weight (Mw) and the isoelectric point (*p*I) of mature protein were 49.9 kDa and 6.4, respectively. Through Simple Modular Architecture Research Tool (SMART), ErPelPL1 consisted of a pectate lyase structure domain, containing a signal peptide (residues 1–26). According to the CAZy database, pectate lyases were distributed in PL1, 2, 3, 9 and 10 families. To explore the evolutionary trace and confirm the subfamilies of ErPelPL1, a phylogenetic tree was constructed. It was found that ErPelPL1 clusters with representative enzymes of subfamily 2 (Fig. 1), thus the ErPelPL1 is a member enzyme belonging to the subfamily 2 of the PL1 family.

**Fig. 2:**
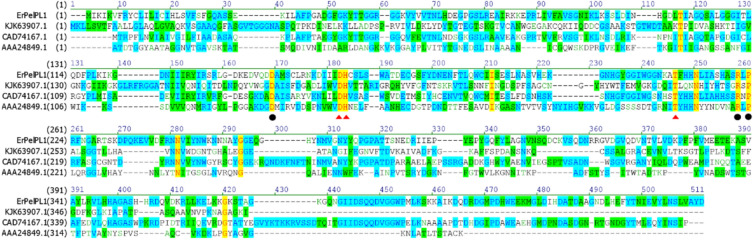
Multiple sequences alignment of ErPelPL1 and partial pectate lyases from the PL1 family. Namely, PelC (*Erwinia chrysanthemii* EC16, AAA24849.1), Pel (*Rhodopirellula baltica* SH1, CAD74167.1) and ApPel1 (*Aspergillus parasiticus*, KJK63907.1). The sequence alignment was performed by Vector-NTI. The three conserved residues coordinated with Ca^2+^ are indicated by black dot. And the potential catalytic residues are marked with red triangle. Background colors represent different similarity: blue (conservative), green (block of similar) and yellow (identical), respectively

**Fig. 3:**
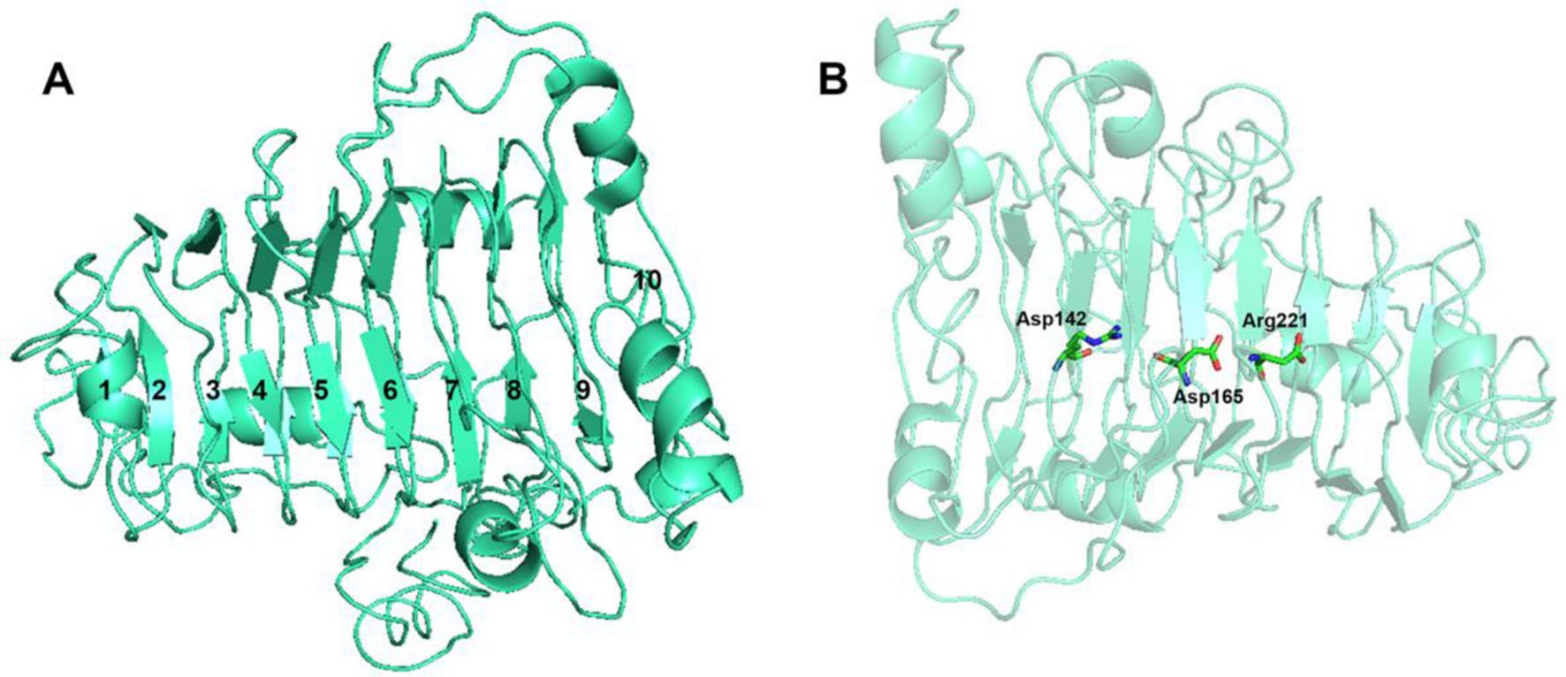
The homology model of ErPelPL1 (**A**) and the key residues for substrate binding (**B**)

At present, all the 11 kinds of structure-resolved pectate lyases from the PL1 family displayed a similar β-helix. Based on multiple sequence alignment analysis with similar protein structures, functionally important amino acid residues and evolutionary trace could be determined (Wang et al. 2018). Therefore, this study performed multiple sequence alignments between ErPelPL1 and other Pels from the PL1 family (Fig. 2). As a result, ErPelPL1 exhibited low sequence identity ranging from 13 to 49%. It shared the highest sequence identity of 49% with Pel (GenBank: CAD74167.1). And it showed the lowest sequence identity of 13% with PelC (GenBank: AAA24849.1) and ApPel1 (GenBank: KJK63907.1). In addition, we explored the key amino acid residues of ErPelPL1 involving catalysis and Ca^2+^-binding residues. As shown in Fig. 2, Asp_169_, Arg_258_ and Pro_260_ were located at the vicinity of the Ca^2+^-binding site, and three potentially catalytic amino acids Asp_182_, His_183_ and Thr_246_ were highly conserved, which have been extensively reported (Henrissat et al. 1995; Lietzke et al. 1996; Yoder et al. 1993). It also revealed key amino acids are usually subjected to intense evolutionary constraints and are strictly conserved. As shown in Fig. 3, the outer convex sheet of the ErPelPL1 contains ten β-strands, and a groove formed by the ten β-strands harbors the catalytic active site as the inner concave sheet.

### Expression and purification of ErPelPL1

**Fig. 4:**
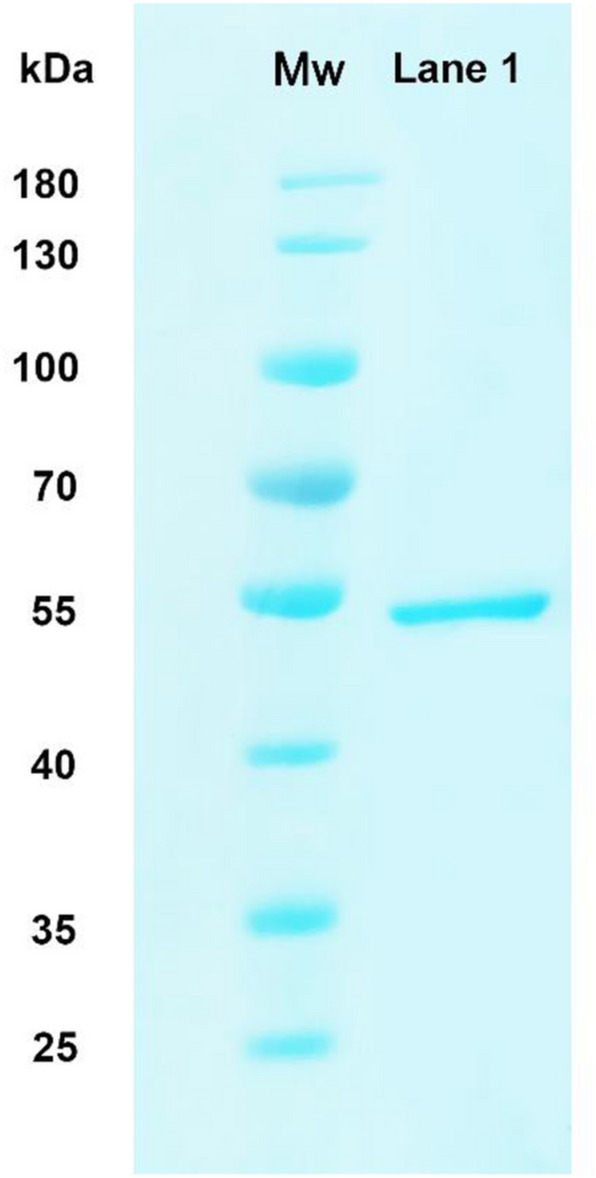
The SDS-PAGE analysis of purified ErPelPL1. Lane left: molecular weight marker (Thermo Scientific, USA); lane 1: purified ErPelPL1

The gene of ErPelPL1 was ligated into pET-21a (+) and then the recombinant plasmid was transformed into *E. coli* BL21 (DE3) for heterologous expression. The recombinant ErPelPL1 was purified by high-affinity Ni-charged resin FF (Ni–NTA Sepharose) prepacked column (GeneScript, Nanjing, China) and analyzed by SDS-PAGE (Fig. 4). The volumetric expression of heterologous protein in the culture was 24.6 mg/L. As shown in Fig. 4, a clear band (about 50 kDa) represented the molecular mass of ErPelPL1 can be observed, which corresponded to the predicted molecular mass of 49.9 kDa. Similarly, the recombinant PelB also has a medium-sized molecular weight of 44 kDa (Wang et al. 2014). Additionally, there are some pectate lyases with a smaller or larger molecular weight. For example, an alkaliphilic and thermostable pectate lyase (Pel-20) from *Actinomadura keratinilytica* strain Cpt20 was a monomer with a molecular mass of 34.1 kDa. And the Pel-15H from *Bacillus* sp. strain KSM-15 has a molecular weight approximately 70 kDa (Ogawa et al. 2000).

### Biochemical characterization of recombinant ErPelPL1

#### Specific activity and kinetic constants

To determine the enzyme activity of ErPelPL1, four conventional substrates (pectin from apple (Pectin A), pectin from citrus peel (Pectin C), polygalacturonic acid (PGA) and polygalacturonic sodium (PG-Na)) were used at 35 ℃ and Tris–HCl (pH 8.0). As a result, the recombinant ErPelPL1 showed higher activity towards PG-Na (59.27 ± 1.83 U/mg) than that to PGA (34.67 ± 1.62 U/mg), Pectin A (1.51 ± 0.45 U/mg) and Pectin C (6.38 ± 1.37 U/mg), respectively. As previously reported, the activity of AsPelA from *Aspergillus luchuensis* var. saitoi towards PGA was 22.2 U/mg, which was lower than ErPelPL1 (Kamijo et al. 2019). In addition, compared with cold-active Pel, ErPelPL1 exhibited a higher activity than that of r-PL D from *Xanthomonas campestris* ACCC 10048 towards PGA (32.0 U/mg) (Yuan et al. 2012). Accordingly, ErPelPL1 was an excellent tool for the degradation of pectic substances, which was conducive to the production of bioactive material POS. In the further experiments, the enzyme kinetics of ErPelPL1 was calculated for the substrates (PG-Na), which was based on hyperbolic regression analysis. The *K*_*m*_ value for ErPelPL1 was 0.161 g/L. The low *K*_*m*_ value of ErPelPL1 for substrate indicates a higher affinity. The *k*_*cat*_ value of ErPelPL1 was 464.56 s^−1^, suggested that this enzyme exhibits higher catalytic efficiency toward substrate. By contrast, the *K*_*m*_ values of PpPel9a and PelB were 0.18 g/L and 1.78 g/L, respectively (Yuan et al. 2019; Wang et al. 2014). The *k*_*cat*_ value of PpPel10a from PL10 family was 202.3 s^−1^ (Zhao et al. 2018). The *K*_*m*_ and *k*_*cat*_ values of BliPelA from alkaliphilic *Bacillus licheniformis* were 0.38 g/L and 193.8 s^−1^, respectively (Zhou et al. 2017).

#### Effect of temperature and pH on ErPelPL1

**Fig. 5:**
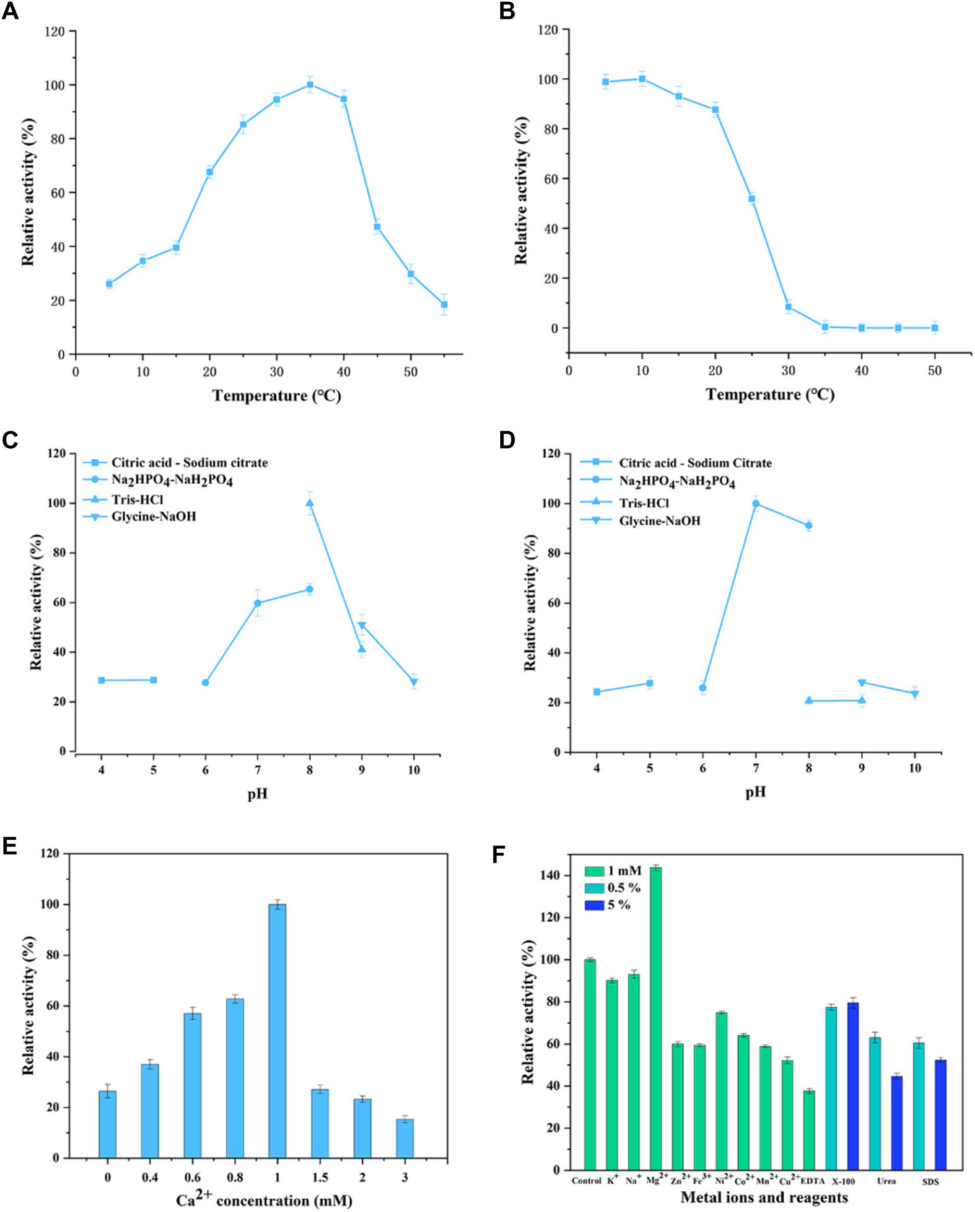
Biochemical characterization of ErPelPL1. **A** The optimal temperature of ErPelPL1. **B** The thermal stability of ErPelPL1. **C** The optimal pH of ErPelPL1. **D** The pH stability of ErPelPL1. **E** The optimal Ca^2+^ concentration of ErPelPL1. **F** The effects of various metal ions and reagents on ErPelPL1. Each value represents the mean of three replicates ± standard deviation. The enzymatic activity without adding metal ions or reagents was designated as 100% served as the control

Generally, mesophilic Pels present the optimal temperature from 40 to 70 °C. Thermo-active Pels always have maximum activity over 60 or 70 °C (Wu et al. 2020). Several researchers defined cold-active enzymes with the optimal temperature around 30 °C (Margesin and Schinner 1991). In addition, the reported cold-active Pels have optimal temperature under 40 °C, and have high activity at low temperature (Wu et al. 2020). In this study, the biochemical characterization results of ErPelPL1 revealed that the optimal temperature was 35 °C and it showed over 85% activity in the range of 25–40 °C (Fig. 5A). The optimal pH of ErPelPL1 was 8.0 (Fig. 5C). It retained about 90% activity incubated with Na_2_HPO_4_–NaH_2_PO_4_ buffer at pH 7.0–8.0 for 12 h (Fig. 5D). In addition, the enzyme could retain more than 80% activity after being incubated at low temperature (10–20 °C) (Fig. 5B). Therefore, ErPelPL1 could be defined as a cold-active pectate lyase.

Compared with other PL1 family Pels, ErPelPL1 exhibited excellent cold-tolerant characteristics. For example, a thermo-alkaline pectate lyase (BliPelA) from an alkaliphilic *Bacillus licheniformis* strain displayed maximum activity at pH 11 and 70 °C (Zhou et al. 2017). The optimal temperature of purified ApPel1 from *Aspergillus parasiticus* was 50 °C, and ApPel1 showed high relative activity from 30 to 60 °C (Yang et al. 2020). The cold-tolerant characteristic of ErPelPL1 was beneficial to several industrial and biotechnological applications, such as the enzymatic preparation of bioactive substance POS and the pretreatment of cotton fiber. However, most reported Pels were classified as mesophilic Pels. At present, there are only three cold-active Pel-encoding genes that have been expressed heterologously and reported (Wu et al. 2020).

To investigate the possible causes of its cold activity, we used GOR4 to predict the secondary structure of ErPelPL1. It has relatively higher contents of irregular curl (52.9%) than those of PEL1 (51%, GenBank number: AXM05364.1) and PLXc (45%, GenBank number: NP_638163), indicating that ErPelPL1 possesses a flexible structure. In addition, ErPelPL1 has relatively higher contents of Met (1.6%) than its mesophilic counterpart (50 °C) from alkaliphilic *Bacillus* sp. N16-5 (1.1%, GenBank number: ACY38198.1) and thermophilic counterpart (90 °C) from *Bacillus* sp. RN1 (1.5%, GenBank number: BAG12908.1), respectively. It is reported that many cold-tolerant enzymes included higher content of Met, which could impart conformational flexibility due to its high degree of freedom and lack of dipole interaction (Yuan et al. 2012). The more flexible structure was conducive to dynamic binding with the substrate at low temperature, thus favoring the occurrence of the enzymatic reaction (Tang et al. 2019). These compositional factors together might lead to the flexible structure of ErPelPL1, consequently allowing the cold-tolerant characteristic. Because cold-active enzymes are less rigid in their structure they are more susceptible to denaturation and deactivation. That might be a reason for the poor stability of ErPelPL1.

In several industrial and biotechnological applications, to prevent the proliferation of undesired microorganisms, retain labile compounds, reduce costs, and save energy, the current trend is to perform these processes at low temperature, such as the production of POS and pretreatment of cotton fabric and pectin waste (Wang et al. 2019; Yuan et al. 2012; Bhatia et al. 2020). However, the commercially available pectinases are highly active at temperatures approximately 50 °C and poorly active at temperatures below 35 °C (Carrasco et al. 2019). Therefore, this remarkable cold-tolerant characteristic indicated ErPelPL1 possesses great potential in several industrial applications that prefers low temperature.

#### Effect of metal ions on ErPelPL1

In further experiments, we introduced the effects of several metal ions and reagents on ErPelPL1 enzyme activity. As shown in Fig. 5E, ErPelPL1 showed maximal activity at the presence of 1 mM CaCl_2_. Based on biochemical characterization data, ErPelPL1 was a cold-tolerant and Ca^2+^-dependent pectate lyase. It also found that ErPelPL1 could retain almost 90% activity incubated with Na^+^ and K^+^. In addition, Zn^2+^, Ni^2+^, Co^2+^, Mn^2+^, Cu^2+^and Fe^3+^ have different inhibitory effects on ErPelPL1 activity. Notably, Mg^2+^ could significantly increase the activity of ErPelPL1. While the presence of 1 mM EDTA (e.g., to chelate the Ca^2+^ present in the reaction mixture) inhibited enzyme activity to a large extent, which indicated ErPelPL1 may be sensitive to these metal ions. Moreover, the enzyme activity was also inhibited by several reagents, such as Triton X-100, urea and SDS (Fig. 5F).

### Action pattern and products distribution

**Fig. 6:**
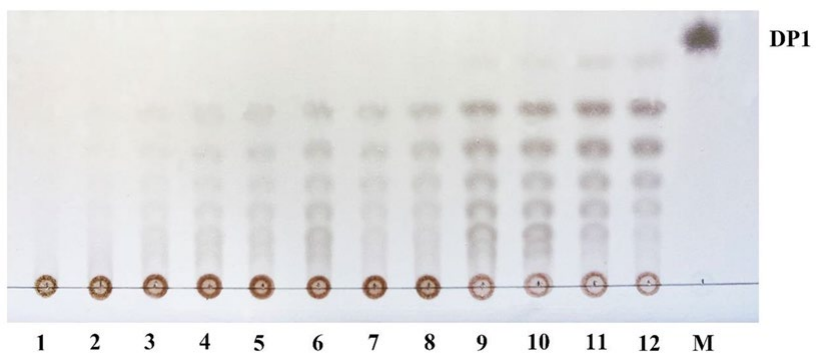
TLC analysis of the ErPelPL1 hydrolyzed products for different times. Lanes 1–12, the samples taken by 5 min, 10 min, 15 min, 30 min, 1 h, 2 h, 4 h, 6 h, 12 h, 24 h, 48 h and 72 h. Lane M, the oligosaccharide standards of d-galacturonic acid sodium salt

**Fig. 7:**
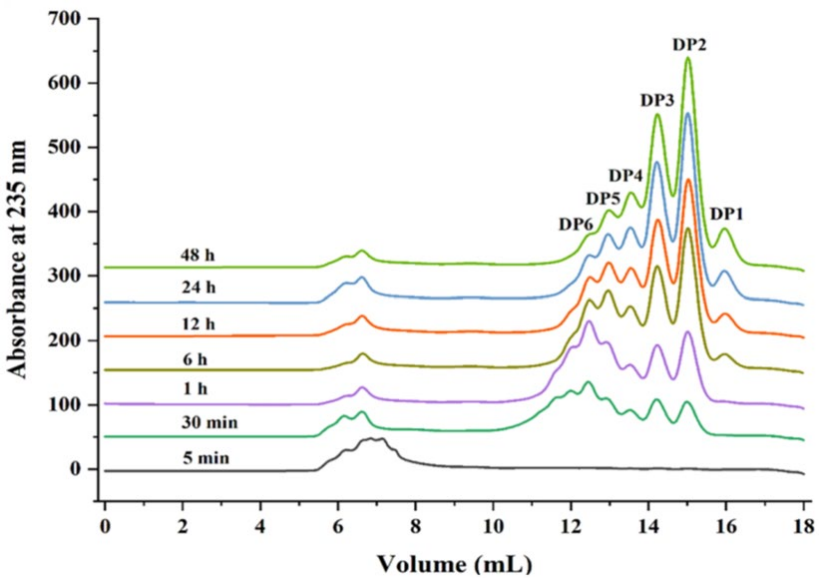
The FPLC analysis of ErPelPL1 degrading products. The reaction times are: 5 min, 30 min, 1 h, 6 h, 12 h, 24 h, 48 h, respectively. The eluents were detected by measuring the absorbance at 235 nm. The elution volumes of the unsaturated monomer, dimer, trimer, tetramer, pentamer and hexamer are 16.25, 15.23, 14.38, 13.61, 13.01, and 12.89 mL

To elucidate the action mode, we combined the thin-layer chromatography (TLC), fast protein liquid chromatography (FPLC) and electrospray ionization mass spectrometry (ESI-MS) using PG-Na as a model substrate to identify the degradation products of the recombinant ErPelPL1. The degradation products for different times (0–72 h) were firstly analyzed by TLC (Fig. 6). As the degrading process continues, the substrate was degraded into oligosaccharides with different degrees of polymerization. Subsequently, the FPLC was applied to further confirm the composition between the starting point and DP1 on TLC. The elution volumes of the unsaturated monomer, dimer, trimer, tetramer, pentamer and hexamer are 16.25, 15.23, 14.38, 13.61, 13.01, and 12.89 mL (Li et al. 2020; Hu et al. 2019). It was found that diverse sizes of oligosaccharides (DP2-6) could be detected at the preliminary stage of degradation (Fig. 7). After 6 h, ErPelPL1 degraded substrate to produce monosaccharide. Interestingly, monosaccharides also appeared in the final stage of the degradation. Therefore, it revealed that oligosaccharides (DP6) were gradually degraded, accompanied by the accumulation of DP1-5 products. As shown in Fig. 7, the final degradation products were mainly DP2-3 unsaturated pectin oligosaccharides. It suggested that ErPelPL1 could cleave the glycosidic bonds within the substrate in an endolytic manner.

**Fig. 8:**
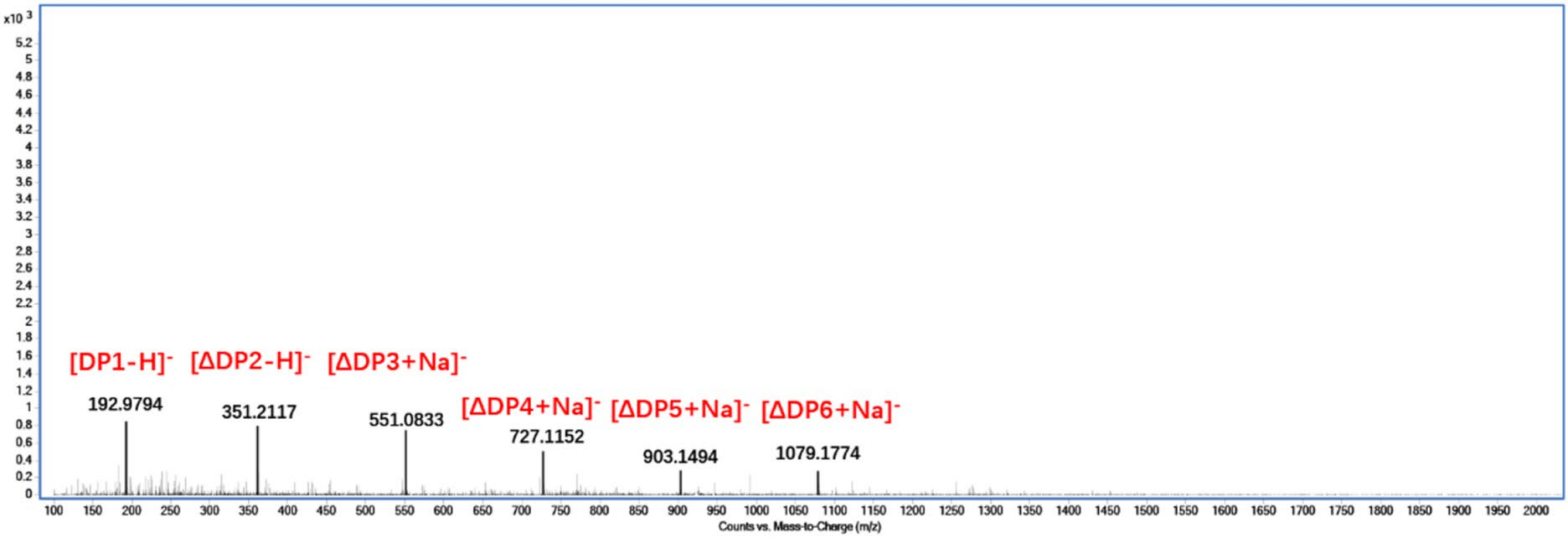
ESI-MS analysis of the degradation products of ErPelPL1 towards polygalacturonic sodium. The mass-to-charge ratios (*m/z*) of oligosaccharide products were detected by using ESI-MS. ΔDP_*n*_ (*n* = 1–6) represents unsaturated oligosaccharides with DP of 1–6

Moreover, we further confirmed the composition of degradation products (unsaturated oligomers) using ESI-MS. As shown in Fig. 8, the ESI-MS showed major intensive peaks at 193, 351, 551, 727, 903 and 1079 *m/z*, indicating that the fractions corresponded to monomer, dimer, trimer, tetramer, pentamer and hexamer, respectively (Wang et al. 2014, 2019; Zhuge et al. 2008). Pectate lyases degrade pectin polymers directly by a β-elimination mechanism, which results in the formation of a double bond between the C4 and C5 atoms of galacturonic acid residues at the non-reducing end by the loss of a water molecular. In conclusion, ErPelPL1 produced a mixture of 4,5-unsaturated oligogalacturonides (including DP1-6), of which DP2-3 dominated. Thus, it confirmed the trans-elimination reaction catalyzing by ErPelPL1 and the recombinant ErPelPL1 could effectively produce galacturonic acid in an endolytic manner. The structure and degree of POS polymerization are closely related to the hydrolytic properties of the pectinase used. Compared with ErPelPL1, several pectate lyases could not degrade oligosaccharides into monosaccharide. For example, the recombinant PL from *Bacillus subtilis* failed to degrade unsaturated trigalacturonic acid and digalacturonic acid to galacturonic acid (Zhuge et al. 2008). In addition, PLHY1 from *Humicola insolens* can only degrade apple peels into pectin oligosaccharides of DP4-6 without smaller oligosaccharides DP1-3 (Wang et al. 2019)), while the ErPelPL1 could degrade the pectin into oligosaccharides with lower DPs and a small fraction of monosaccharide. According to the products distribution, the ErPelPL1 seemed to degrade the pectin more thoroughly and efficiently and it is suitable for preparation of pectin oligosaccharides with low DPs such as disaccharides and trisaccharides. In addition, the monosaccharides’ ratio could be restricted at a relatively low level under properly controlled conditions. Perhaps a mixture enzyme system of ErPelPL1 and PLHY1 can effectively degrade pectin into POS, which is helpful to full utilization of pectin waste. This study provides a new candidate enzyme for production of POS. Enzymatic pectin hydrolysis to obtain POS is a crucial area of research, as this method offers several advantages, namely substrate specificity, mild hydrolysis conditions, low cost, and environmental safety. Therefore, unsaturated pectin oligosaccharides can be economically produced by utilizing pectate lyase and low-cost wastes (apple, orange peels) from the agro-food industry.

## Conclusion

Herein, a novel cold-tolerant Pel from the subfamily 2 of PL1 family was firstly cloned, purified, and characterized, which is effective at normal room temperature. The recombinant ErPelPL1 showed strong Ca^2+^ dependence and excellent cold-tolerant property, which could be beneficial for several industrial and biotechnological applications, such as the production of POS, the pretreatment of cotton fabric and animal feed. Through degradation products analysis, ErPelPL1 endolytically degraded pectic substances into oligosaccharides ranging from monosaccharide to hexasaccharide (DP1-6). Therefore, ErPelPL1 has potential applications in producing 4,5-unsaturated POS for biological activity research. This study also provides a valuable candidate for cold-tolerant Pels.

### Supplementary Information

**Additional file 1: Table S1.** The primers for cloning the gene of pectate lyase ErPelPL1.

## Data Availability

All data supporting the findings of this study are available in the article, supporting information, or upon request from the corresponding author.

## Abbreviations

TLC: Thin-layer chromatography
FPLC: Fast protein liquid chromatography
ESI-MS: Electrospray ionization mass spectrometry
DP: Degree of polymerization
POS: Pectin oligosaccharide
Pel: Pectate lyase
HM: High-methoxyl
LM: Low-methoxyl
PUL: Polysaccharide utilization loci
PL: Polysaccharide lyase
Pectin A: Pectin from apple
Pectin C: Pectin from citrus peel
PGA: Polygalacturonic acid
PG-Na: Polygalacturonic sodium
DNS: 3,5-Dinitrosalicylic acid
