# Supplementary material for: Elucidating the degradation pattern of a new cold-tolerant pectate lyase used for efficient preparation of pectin oligosaccharides
Source: Bioresour Bioprocess. 2021 Dec 4;8(1):121. doi: 10.1186/s40643-021-00475-2 (PMC10992097; doi:10.1186/s40643-021-00475-2)
Supplement: Supplementary file 1 — Additional file 1: Table S1. The primers for cloning the gene of pectate lyase ErPelPL1. [file 40643_2021_475_MOESM1_ESM.docx]

**Table S1** The primers for cloning the gene of pectate lyase ErPelPL1

| Primer | Sequence |
| --- | --- |
| ErPelPL1F | CATATGAGCAGCGAAAAAATTCTGGC |
| ErPelPL1R | CTCGAGATCATACGCCACCAGGCTGT |
